# DNA plasmid HIV vaccine design, number of doses, participant gender, and body mass index affect T-cell responses across HIV vaccine clinical trials

**DOI:** 10.1186/1742-4690-9-S2-P133

**Published:** 2012-09-13

**Authors:** C Morgan, X Jin, X Yu, S De Rosa, J Kublin, B Metch, M Keefer, H NIAID

**Affiliations:** 1 HIV Vaccine Trials Network / Fred Hutchinson Cancer Research Center, Seattle, WA, USA; 2 University of Rochester Medical Center, Rochester, NY, USA

## Background

Numerous studies have evaluated individual DNA plasmid vaccines. We evaluated data from 10 HIV vaccine clinical trials that utilized common, validated immunogenicity assays to objectively investigate factors that influence DNA plasmid-induced T-cell responses.

## Methods

This retrospective analysis included data from 1218 healthy, HIV-1 uninfected adults enrolled in 10 DNA HIV vaccine clinical trials conducted within the HIV Vaccine Trials Network. HIV-specific T-cell responses from peripheral blood mononuclear cells were measured using validated IFN-γ ELISpot and intracellular cytokine staining assays. The effects of DNA vaccine HIV antigens, number of doses, gender, body mass index (BMI), and age were evaluated.

## Results

When plasmids expressing Gag, Env and Pol were co-administered, the highest T-cell response rate was against Env (38.1%) and much less to Gag (4.6%) or Pol (4.5%). Comparing 2, 3, and 4 DNA injections, 3 vaccinations compared to 2 improved the magnitude of Env-specific CD8+ T-cell response (p=0.048), but not CD4+ T-cell responses, and a 4th vaccination had no additional effect. HIV-specific CD4+ T-cell response rates were higher in females (50.9%) than in males (27.7%, p=0.0002). Having lower BMI (p=0.025) was independently associated with higher HIV-specific CD4+ T-cell response rates. There were no significant differences in HIV-specific CD8+ T-cell response frequencies by gender or BMI. Lower HIV-specific CD4+ T-cell responses were seen with the 18-20 (36.4%) and 41-50 (23.3%) age groups compared to the 21-30 (44.0%) and 31-40 (41.%) groups, but these differences were not significant (p=0.0565) and no differences were seen in HIV-specific CD8+ T-cell responses by age.

## Conclusion

Pooled data across DNA HIV vaccine clinical trials indicate that plasmid inserts, number of doses, gender, and BMI can affect T-cell responses. These factors should be considered in vaccine research.

